# Dr. Alderson's Claim to Priority of Baron Larrey

**Published:** 1817-07

**Authors:** John Higson

**Affiliations:** House-Surgeon of the General Infirmary, Hull.


*For the London Medical and Physical Journal.*

*Dr. Alderson's Claim to Priority of Baron Larrey;* by John Higson, Esq. House-Surgeon of the General Infirmary, Hull.

IN your valuable Journal for last January, you have inserted a report on a Memoir read by Baron Larrey, at the Royal Institution of France, in which report the Baron is complimented on his discovery of a method of treating wounds of the chest from balls.

In the *third* volume of your work, page 353, your readers will find a case successfully treated in the same manner, under the direction of Dr. Alderson, senior physician to this institution, in the year 1799,—so that Baron Larrey is not the inventor of a peculiar mode of treating such wounds.

*Hull; May* 31, 1817.

